# Public Information-Seeking Behavior in Primary Care: A National Case Study of Türkiye

**DOI:** 10.7759/cureus.111486

**Published:** 2026-06-25

**Authors:** Muhammed Inan, Cenk Aypak

**Affiliations:** 1 Department of Family Medicine, University of Health Sciences Ankara Etlik City Hospital, Ankara, TUR

**Keywords:** family medicine, google trends, health status disparities, search engine, telemedicine

## Abstract

Objective

This study utilizes digital epidemiology to examine public engagement with family medicine in Türkiye, aiming to inform strategies for optimizing primary care delivery and reducing health disparities.

Method

This observational study analyzed public interest in family medicine in Türkiye using Google Trends data between January 2018 and April 2025, following the STROBE guidelines. A total of 56 keywords were selected based on expert consensus and search volume assessment, excluding redundant or low-volume queries. Statistical analyses, including Mann-Kendall (MK) trend tests, one-way analysis of variance (ANOVA), and OLS regression, were conducted using Python (v3.12; Python Software Foundation, Delaware, USA). To control for type I error across multiple comparisons, all p-values were adjusted using the Benjamini-Hochberg false discovery rate (FDR) procedure. Practical significance was further evaluated using Eta-squared (η²) and Cohen’s d effect sizes, and model robustness was verified via regression diagnostics (Durbin-Watson and Shapiro-Wilk tests).

Results

Long-term interest in family medicine services demonstrated significant growth, with “what is a family physician?” (τ = 0.759, pFDR < 0.001) and “family health center” (τ = 0.558, pFDR < 0.001) emerging as the most prominent upward trends. Seasonal analysis revealed significant variation for “family physician” (η² = 0.232, pFDR = 0.003), with peaks in winter and fall, aligning with respiratory illness cycles. Impact analysis of the COVID-19 pandemic confirmed a sustained transition to digital platforms, particularly for the “Central Physician Appointment System” (new-normal Cohen’s d = 5.05, pFDR < 0.001). Regionally, while metropolitan centers dominated general appointment searches, specific healthcare needs were highlighted by unique regional patterns, such as the high search intensity for “family physician on-call” in Ordu.

Conclusion

Although public interest in family medicine has steadily increased, significantly accelerated by the pandemic's shift to digital platforms, information gaps regarding the scope of services remain. Furthermore, geographic disparities in search behavior point to a potential "digital shadow" in rural areas, suggesting localized informational deficits. These digital footprints provide hypothesis-generating insights that health administrators may consider as supplementary tools for regional planning and guiding digital literacy initiatives in underserved regions.

## Introduction

Family medicine serves as the cornerstone of primary healthcare systems worldwide, offering comprehensive, continuous, and person-centered care. In Türkiye, the Family Medicine System - fully implemented by 2010 - restructured primary care by assigning each physician to a registered population, focusing on prevention, chronic disease management, and health promotion [1]. While each physician ideally serves around 3,500 patients [2], this varies by geography and demand [3]. Despite expanding access, the system faces challenges, such as workforce shortages [4] and service integration issues [1]. Understanding how individuals seek information about family medicine is vital for improving accessibility and responsiveness.

Türkiye’s digital health infrastructure, particularly Merkezi Hekim Randevu Sistemi (MHRS; Central Physician Appointment System) and e-nabız (national e-health platform), plays a critical role in accessing family medicine. However, digital disparities - especially in rural areas - exacerbate health inequities. Unequal internet access and digital literacy create vulnerabilities that limit equitable care engagement.

Although Google Trends has been applied across various clinical domains, including infectious disease surveillance and oncology [5,6], its use in the context of family medicine information seeking has not been previously reported, to our knowledge. Research consistently shows that patients search for health information online before contacting a provider [7,8]. This pre-consultation behavior - information seeking that precedes the decision to access care - shapes which services patients seek and how they engage with the health system. Search data therefore capture knowledge gaps and navigational barriers that clinical records cannot [8,9]. This exploratory research fills that gap by analyzing search behavior from 2018 to 2025, integrating health services research with digital epidemiology. The comparison between family medicine and internal medicine search trends is hypothesis-driven, based on the premise that internal medicine is a longer-established specialty, whereas family medicine represents a relatively newer primary care discipline in Türkiye. The remaining analyses - trend identification, seasonal patterns, and regional mapping - are descriptive and hypothesis-generating. Objectives include identifying long-term and seasonal trends, uncovering thematic patterns in search terms, and comparing public interest in family versus internal medicine, with the aim of informing health policy and reducing regional disparities in primary care engagement.

## Materials and methods

Data collection

Google Trends data were collected for 56 keywords related to family medicine in Türkiye between January 1, 2018, and April 30, 2025. All keywords were queried in Turkish to reflect the language used by the target population in routine online searches. Keywords such as "family medicine," "family medicine appointment," "health center," and "what is a family physician" were selected based on expert consultation with two family medicine specialists and preliminary examination of trend data for relevant terms. The process began with 71 candidate terms, refined to 56 keywords (54 evaluated in the trend, seasonal, and pandemic-phase analyses, plus 2 comparative terms used only in the ordinary least squares (OLS) regression comparing family medicine and internal medicine search trajectories) based on search volume, relevance, and the exclusion of sparse or redundant queries (see Appendices). Duplicate terms (e.g., "family physician" vs. "family doctor") were retained to reflect real-world search behavior. Both specialists reached consensus on all final keyword selections without disagreement.

Search intensity data - reported as normalized relative search volumes (RSV) from 0 to 100 - were obtained via https://trends.google.com with location filtering set to Türkiye. Monthly RSV data were collected for each keyword at both national and city levels. The data are anonymous, aggregated, and publicly available, raising no ethical concerns.

Data preprocessing

Monthly data resulted in 88 monthly time points per keyword (Jan 2018-Apr 2025). RSV values labeled “<1” were coded as 0, while missing or null values were also set to 0. The dataset was divided into pre-COVID-19 (Jan 2018-Feb 2020), acute-COVID (Mar 2020-Dec 2021), and new-normal (Jan 2022-Apr 2025) periods. For seasonal analysis, seasons were defined as winter (Dec-Feb), spring (Mar-May), summer (Jun-Aug), and fall (Sep-Nov).

Statistical analysis

Statistical analyses were performed using Python 3.12 (Python Software Foundation; Delaware, USA) with the pandas 2.2, NumPy 1.26, SciPy 1.12, and statsmodels 0.14 libraries.

Monotonic trends were assessed using the Mann-Kendall (MK) test and supported by Spearman’s rank correlation coefficient (ρ). To reduce the risk of type I error due to multiple comparisons, all reported p-values were adjusted using the Benjamini-Hochberg false discovery rate (FDR) procedure, and statistical significance was defined as FDR-adjusted p < 0.05. RSV values reported as "<1" were initially coded as 0.5. A sensitivity analysis recoding these values as 0 yielded materially consistent results; the final analyses therefore used 0 to avoid introducing arbitrary non-zero estimates into sparse data.

Seasonal differences were evaluated using one-way analysis of variance (ANOVA), and effect sizes were quantified using eta-squared (η²). Differences between pandemic periods were examined using independent Welch’s t-tests, with effect sizes reported as Cohen’s d.

To compare temporal trends between the “family medicine specialist” and “internal medicine specialist” groups, an OLS regression model was applied. Temporal autocorrelation was assessed using the Durbin-Watson statistic, and residual normality was evaluated using the Shapiro-Wilk test.

## Results

General characteristics and long-term trends

Of the 54 analyzed keywords, 26 (48.1%) showed a statistically significant monotonic trend over the January 2018-April 2025 period following Benjamini-Hochberg FDR correction (Table 1).

The strongest continuous upward trends were observed for "what is a family physician?" (τ = 0.759, pFDR < 0.001), "family physician's salary" (τ = 0.668, pFDR < 0.001), and "can a family physician prescribe medication?" (τ = 0.567, pFDR < 0.001).

Four keywords showed significant downward trends: "family medicine specialization" (τ = -0.218), "search family physician" (τ = -0.217), "family physician address" (τ = -0.330), and the abbreviated query "FHC appointment" (τ = -0.364), all pFDR < 0.05.

**Table 1:** Long-Term Search Trends (2018-2025) Trend analyses were conducted covering the period from January 2018 to April 2025. Monotonic trends were assessed using the Mann-Kendall (MK) test and supported by Spearman’s rank correlation coefficient (ρ). To control for multiple comparisons, all p-values were adjusted using the Benjamini-Hochberg false discovery rate (FDR) procedure. Statistical significance was defined as pFDR < 0.05. MK: Mann-Kendall; FDR: false discovery rate; MHRS: Central Physician Appointment System; FHC: family health center

Keyword	MK Tau (τ)	MK p (FDR)	Spearman Rho (ρ)	Spearman p (FDR)
What is a family physician?	0.759	<0.001	0.921	<0.001
Family physician's salary	0.668	<0.001	0.862	<0.001
Can a family physician prescribe medication?	0.567	<0.001	0.699	<0.001
Family health center	0.558	<0.001	0.736	<0.001
What does a family physician do?	0.515	<0.001	0.668	<0.001
Family physician health report	0.506	<0.001	0.717	<0.001
Family physician specialist	0.467	<0.001	0.625	<0.001
MHRS (Central Physician Appointment System) appointment	0.439	<0.001	0.674	<0.001
Family physician MHRS (Central Physician Appointment System) appointment	0.4	<0.001	0.637	<0.001
MHRS (Central Physician Appointment System)	0.358	<0.001	0.611	<0.001
Family physician e-Devlet (electronic government gateway)	0.357	<0.001	0.495	<0.001
How to get a family physician appointment?	0.345	<0.001	0.453	<0.001
Health center working hours	0.327	<0.001	0.468	<0.001
Health center	0.304	<0.001	0.459	<0.001
Family physician	0.299	<0.001	0.423	<0.001
How to become a family physician?	0.257	0.002	0.368	0.001
Family health center (FHC) appointment	0.244	0.003	0.416	<0.001
Family physician examination	0.243	0.003	0.361	0.002
Family physician on-call	0.228	0.017	0.286	0.016
Family medicine blood work	0.21	0.011	0.301	0.011
Family medicine	0.195	0.019	0.318	0.007
Nearest family physician	0.182	0.045	0.261	0.028
Search family physician	-0.217	0.027	-0.266	0.027
Family medicine specialization	-0.218	0.022	-0.273	0.023
Family physician address	-0.33	<0.001	-0.467	<0.001
FHC appointment (abbreviated query)	-0.364	<0.001	-0.513	<0.001

The remaining 28 keywords (51.9%) did not reach significance after FDR correction (|τ| range: 0.01-0.19; pFDR range: 0.06-0.89), including several appointment and logistics queries such as "family physician appointment" (τ = 0.116, pFDR = 0.180), "family doctor appointment" (τ = 0.186, pFDR = 0.062), "family physician sick-leave report" (τ = 0.167, pFDR = 0.101), and "family physician COVID test" (τ = -0.041, pFDR = 0.673). One borderline case was noted: "family physician e-Nabız" showed a significant Spearman's correlation (ρ = 0.263, pFDR = 0.028) but did not reach significance on the MK test (τ = 0.175, pFDR = 0.057).

A total of 15 keywords were excluded from comparative analyses due to persistently low search volume. These keywords included queries related to cancer screening, diabetes monitoring, heel prick testing, smear testing, chronic disease management, telehealth, tele-examination, newborn registration, vaccination appointments, online appointments, health center appointments, finding a family physician, family physician verification with national ID, and out-of-hours availability. The near-total absence of search activity for these terms across the study period represented a notable null pattern, suggesting that specific procedure-level primary care information needs did not translate into sustained national search behavior.

Seasonal variations

One-way ANOVA revealed significant seasonal differences (p < 0.05) for two keywords (Table 2). "Family physician" displayed the strongest seasonal pattern (F = 8.456, pFDR = 0.003, η² = 0.232), with means highest in fall (69.0) and winter (65.9) and lowest in spring (55.2). A second, smaller effect (η² = 0.171, pFDR = 0.033) was observed for a salary-related query. The remaining 52 keywords showed no significant seasonal variation after correction.

**Table 2:** Seasonal Variations in Search Intensity Seasonal differences in normalized search volumes were evaluated using one-way analysis of variance (ANOVA). Effect sizes were quantified using Eta-squared (η²). To control for multiple comparisons, all p-values were adjusted using the Benjamini-Hochberg false discovery rate (FDR) procedure. Statistical significance was defined as pFDR < 0.05. ANOVA: analysis of variance; FDR: false discovery rate

Keyword	Winter	Spring	Summer	Fall	ANOVA F	η²	p (FDR)
Family physician	65.9	55.2	60.8	69	8.456	0.232	0.003
Family physician’s salary	65.4	49.7	62.6	62.9	5.765	0.171	0.033

Impact of the COVID-19 pandemic phases

Of the 54 analyzed keywords, 40 had sufficient non-zero search volume across all three pandemic phases (pre-COVID, acute-COVID, new-normal) to support valid Welch's t-test/Cohen's d estimation. Among these, 29 (72.5% of testable keywords) showed a statistically significant difference in at least one phase comparison after FDR correction (Table 3). The largest sustained shifts (pre-COVID → new-normal) were seen for "MHRS (Central Physician Appointment System) appointment" (d = 5.05, pFDR < 0.001), "what is a family physician?" (d = 3.81, pFDR < 0.001), and "family health center" (d = 3.59, pFDR < 0.001), indicating a durable transition toward digital primary-care engagement that persisted well beyond the acute pandemic phase rather than reverting afterward.

**Table 3:** Shifts in Search Intensity Across Pandemic Phases Mean normalized search volumes are reported for pre-COVID, acute-COVID, and new-normal phases. Differences between phases were analyzed using independent Welch's t-tests. Effect sizes were quantified using Cohen's d. To control for multiple comparisons, all p-values were adjusted using the Benjamini-Hochberg false discovery rate (FDR) procedure. Statistical significance was defined as pFDR < 0.05. FDR: false discovery rate; MHRS: Central Physician Appointment System; FHC: family health center

Keyword	Pre-COVID	Acute-COVID	New-normal	d (Pre→acute)	p (FDR)	d (Pre→new-normal)	p (FDR)
MHRS (Central Physician Appointment System) appointment	28.7	49.3	69.6	1.01	0.013	5.05	<0.001
What is a family physician?	31.4	44.5	68.8	1.36	<0.001	3.81	<0.001
Family health center	43.2	70.3	71.5	2.17	<0.001	3.59	<0.001
MHRS (Central Physician Appointment System)	29.5	38.1	43.2	0.58	0.146	3.29	<0.001
Family physician MHRS (Central Physician Appointment System) appointment	19	39.5	46.1	1.23	0.003	3.11	<0.001
Family physician's salary	20.7	36.2	59.2	1.47	<0.001	3.06	<0.001
What does a family physician do?	0	6.2	49.8	0.57	0.151	2.28	<0.001
Health center	47.3	70.2	61.4	1.97	<0.001	2.07	<0.001
Family physician specialist	5.5	15.7	52.6	0.47	0.204	2.06	<0.001
Family physician health report	30.9	29.5	53.5	-0.16	0.701	1.96	<0.001
Family physician e-Devlet	11.2	52.9	59.6	1.72	<0.001	1.96	<0.001
Can a family physician prescribe medication?	0	4.8	41.4	0.46	0.249	1.85	<0.001
FHC appointment	52.8	47.7	34.5	-0.31	0.409	-1.57	<0.001
Family health center appointment	28.8	41.2	39.1	0.82	0.043	1.46	<0.001
How to get a family physician appointment?	0	12.7	30.2	0.68	0.096	1.21	<0.001
Family physician address	49.3	47	26.3	-0.1	0.767	-1.12	<0.001
Family physician e-Nabız	4.3	34.7	30.8	1.13	0.005	1.1	<0.001
Family medicine	38.3	57.8	46.1	1.42	<0.001	1.1	<0.001
How to become a family physician?	21.2	38.3	44.4	0.66	0.077	1	<0.001
Family doctor e-Nabız	2.2	43.7	25.4	1.72	<0.001	0.97	<0.001
Family physician	56.8	62.7	66.4	0.63	0.096	0.96	<0.001
Family medicine specialization	25	4.1	3.7	-0.79	0.022	-0.92	0.008
Health center appointment	35	51.4	42.8	1	0.013	0.91	<0.001
Health center working hours	60	61.5	68.5	0.12	0.751	0.76	0.009
Nearest family physician	11.1	29.8	31.2	0.64	0.088	0.7	0.01
Family medicine blood work	49.6	42.8	61.1	-0.32	0.409	0.63	0.024
Family physician appointment	54.6	59.2	62	0.3	0.409	0.63	0.016
Family physician on-call	3.8	6	19	0.11	0.767	0.58	0.022
Family physician COVID test	0	20.9	3.8	0.86	0.038	0.35	0.123

Three keywords moved in a negative direction across the pandemic: "FHC appointment" (abbreviated form; d = -1.57), "family physician address" (d = -1.12), and "family medicine specialization" (d = -0.92, with an already-negative shift during the acute phase, d = -0.79). These mirror the same three keywords identified as long-term decliners in Table 1, reinforcing internal consistency between the trend and pandemic-phase analyses rather than reflecting isolated artifacts.

Eleven keywords (27.5% of testable keywords) showed no significant difference in either comparison, including "family medicine appointment," "family doctor," "family physician duties," and "family physician fee" (all pFDR > 0.05 for both acute and new-normal comparisons; |d| ≤ 0.59).

Trend comparison between family medicine and internal medicine specialties

**Figure 1:**
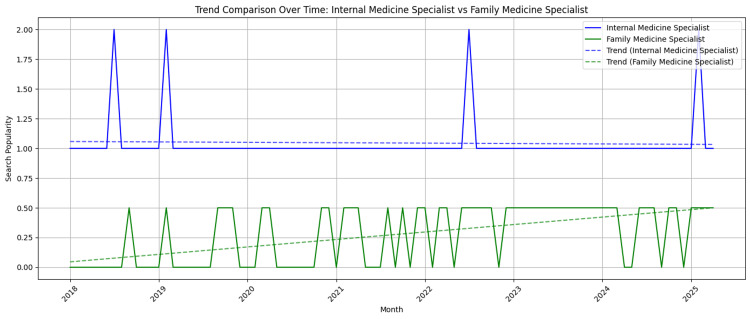
Trend Comparison Between Family Medicine Specialists and Internal Medicine Specialists

An OLS regression model compared the longitudinal search trajectories of “family medicine specialist” and “internal medicine specialist.” Regression diagnostics confirmed model robustness: the Durbin-Watson statistic (1.916) indicated no significant temporal autocorrelation, and the Shapiro-Wilk test (p = 0.517) confirmed the normal distribution of the residuals. The interaction term between time and specialty group was statistically significant (coefficient = 0.173, 95% confidence interval (CI): 0.054 to 0.292, p = 0.005), confirming that search volume for family medicine specialists accelerated at a significantly steeper rate than that of internal medicine (Figure 1).

Search intensity across cities

**Figure 2:**
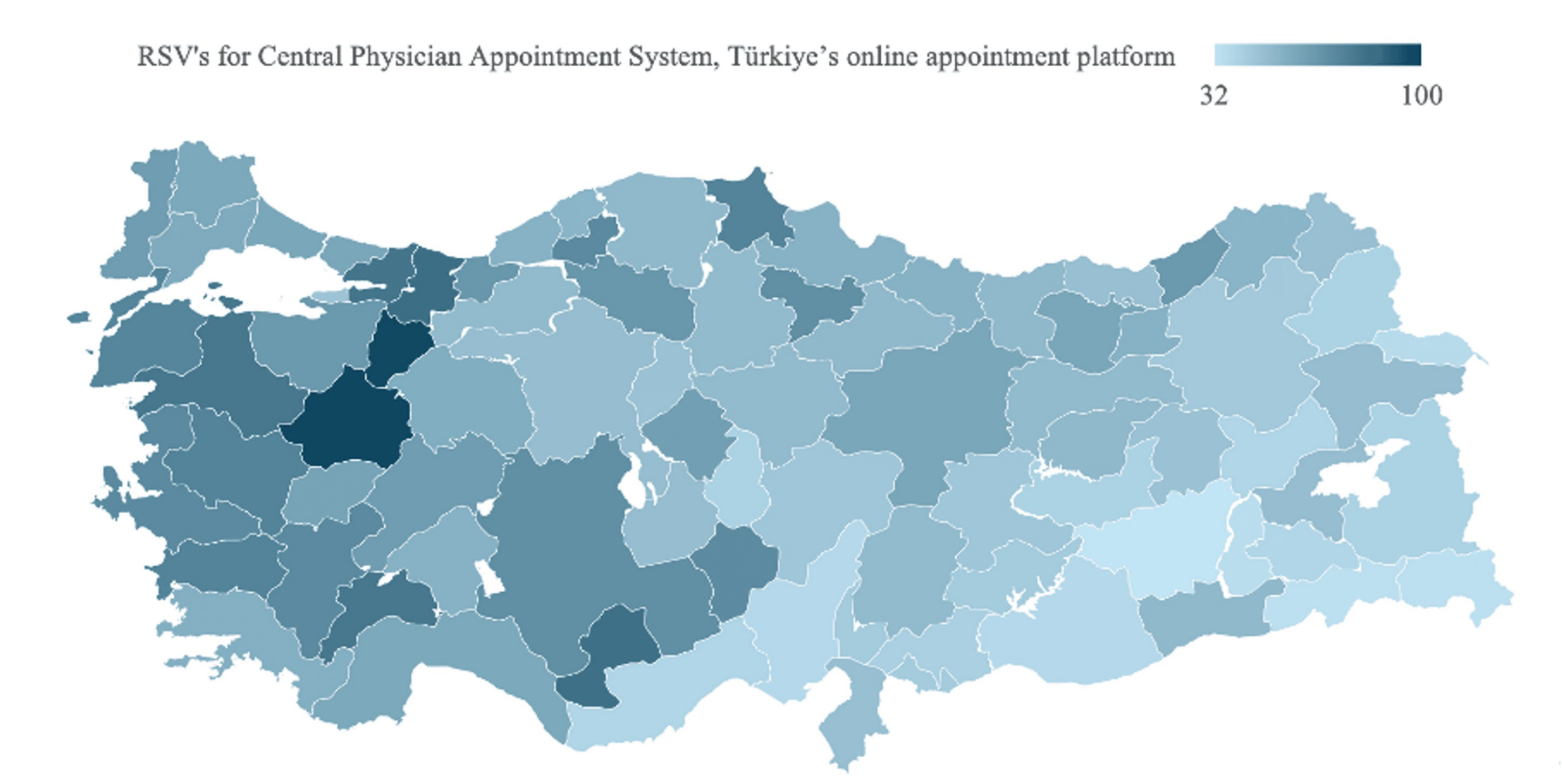
Geographical Distribution of Relative Search Volumes (RSVs) for Central Physician Appointment System, Türkiye’s Online Appointment Platform

The analysis of search intensity for keywords across Turkish cities revealed distinct regional preferences. For "family physician on-call," Ordu exhibited the highest intensity (100.0), followed by Sakarya, Mersin, and Eskişehir (each 40.0), with İzmir showing 20.0 intensity. For "family physician home care," Yalova led with an intensity of 100.0, followed by Sakarya (37.0), while İzmir, İstanbul, and Ankara recorded minimal intensity (0.5). The keyword "Central Physician Appointment System online appointment" was most intensely searched in Kütahya (100.0) and Bilecik (99.0), with Sakarya (83.0), Karaman (82.0), and Kocaeli (80.0) also ranking high (See Figure 2 for geographical distribution of relative search volumes for Central Physician Appointment System, Türkiye’s online appointment platform). Additionally, for "family physician drug prescription," Afyonkarahisar showed the highest intensity (100.0), followed by Konya (20.0), with Ankara, İzmir, and İstanbul at low intensity (0.5). Finally, "switch family physician" was most prominent in Kırklareli (100.0), İstanbul (92.0), Rize (85.0), Tekirdağ (71.0), and Ankara (71.0).

## Discussion

This study is the first to present a comprehensive exploration of public interest in family medicine using digital search behavior, specifically Google Trends data. It is important to acknowledge that Google Trends data are indicators of information-seeking interest, not proxies for healthcare utilization or clinical need. By analyzing 56 family medicine-related keywords between 2018 and 2025, the study identifies long-term trends and seasonal patterns that provide novel insights into public engagement with primary care services, specifically in Türkiye. This research contributes to the growing field of digital epidemiology and offers policy-relevant evidence to support more equitable and responsive primary care delivery.

A key finding is the rising interest in the roles and responsibilities of family physicians. Significant upward trends in searches such as “what is a family physician?” and “family physician’s salary” reflect a shift in public curiosity and awareness. This change may be attributed to national health reforms, increased media coverage, and public health campaigns aimed at strengthening the visibility of family medicine. Moreover, the COVID-19 pandemic likely acted as a catalyst for health-seeking behavior, leading to greater public reliance on digital platforms and increased demand for accessible healthcare information. The results demonstrate an ongoing evolution in public perception, with family physicians increasingly recognized as vital components of Türkiye’s health system. The significant upward trend in searches for “family physician salary” and “how to become a family physician” should be interpreted with nuance, as these queries may reflect a combination of public curiosity about the healthcare workforce, intra-professional interest among medical students and practitioners, attention to economic developments within the primary care sector, and increased visibility stemming from media coverage or policy discussions rather than genuine public uncertainty alone.

Seasonal variation in search behavior was another important pattern. The term "family physician" showed the strongest seasonal pattern, with search volumes peaking in fall and winter (Table 2), aligning with seasonal spikes in respiratory infections and chronic illness exacerbations [6,10]. This observation supports previous studies linking increased healthcare demand during colder months with flu season and other communicable diseases [11]. The seasonal peaks in searches for "family physician" during winter and fall highlight opportunities for public health workers to collaborate with family health centers to manage increased demand, ensuring equitable access to care through community-based outreach and digital navigation support.

Post-COVID-19 trends are especially noteworthy. Increased search volumes for "health center" (d = +2.07) and "family health center" (d = +3.59) suggest that the pandemic not only raised health awareness but also accelerated the transition toward digital engagement with healthcare services. The normalization of digital appointment booking systems and reliance on platforms such as the Central Physician Appointment System and e-nabız reflect broader shifts toward digital health infrastructure. However, the limited upward trend in COVID-specific searches such as “family physician COVID test” (τ = -0.041, pFDR = 0.673) suggests that family physicians were not the primary point of care for COVID-19 diagnosis or management [12]. This is consistent with the referral of COVID-related services to secondary and tertiary hospitals during the crisis.

Interestingly, search interest in “family medicine specialist” grew more rapidly than for “internal medicine specialist,” indicating a shift in public recognition of family medicine as a standalone specialty. This may be due to increasing responsibilities taken on by family physicians, particularly in chronic disease management, which traditionally fell within internal medicine’s scope. These findings reflect global trends seen in strong primary care systems such as those in the Netherlands and the UK, where family physicians are pivotal in prevention, continuity of care, and chronic disease management [13]. However, unlike systems with structured gatekeeping (e.g., the UK), Türkiye lacks strict referral mechanisms, which may contribute to continued ambiguity regarding family physicians’ roles. The popularity of the search “what is a family physician?” suggests the need for better public communication and branding of primary care services.

Increased search frequency for keywords such as “can a family physician prescribe medication?” and “family physician health report” also reveals public uncertainty about the scope of services provided by family physicians [14]. These queries indicate a lack of clarity around practice boundaries and reinforce the need for targeted health education initiatives. Similar informational trends have been observed in other countries undergoing primary care reform [5,15], suggesting that digital search behavior can function as a proxy for informational deficits in healthcare systems.

In contrast to these upward trends, some keywords, such as “family physician address” and “family health center appointment,” exhibited declining search patterns. This is somewhat surprising given the broader shift to digital engagement. One possible explanation is the increasing dominance of centralized digital systems, such as the Central Physician Appointment System, which offer integrated functionalities for booking appointments and locating providers [16]. Türkiye’s unique digital infrastructure may reduce the need for manual searches for individual addresses, diverging from patterns observed in other countries where location-based queries increase alongside digital health adoption [17]. Users may also have migrated to in-app search functions within MHRS, reducing general web queries for provider addresses - a shift Google Trends cannot capture. While this efficiency may reflect successful platform integration, it also raises concerns about the digital divide. Populations with limited internet access or low digital literacy may face greater barriers to navigating centralized systems, potentially exacerbating health inequities.

The trend data revealed a consistent thematic structure in how the public searches for family medicine information. Role-definition queries - "what is a family physician?," "what does a family physician do?," "can a family physician prescribe medication?" - shared similarly strong upward trajectories (τ > 0.50) and moved together across pandemic phases [9]. Platform-related queries, such as MHRS appointment and family physician MHRS appointment, showed a comparable co-movement pattern, particularly in the new-normal period (Table 3). In contrast, emergency-adjacent terms, such as "family physician on-call," showed the weakest significant upward trend among all analyzed keywords (τ = 0.228) throughout the study period. This pattern suggests that the public does not strongly associate family physicians with urgent or emergency care needs, which is consistent with their defined role in the Turkish primary care system [13].

Following these findings, regional variation in search patterns was briefly considered in a descriptive manner. Differences in search intensity across regions were observed, which may be related to variation in healthcare access and engagement. For instance, the high search intensity for “family physician on-call” in Ordu (100.0) may indicate unmet emergency care needs in rural areas, where hospital access is limited due to geographic isolation and dispersed populations [18]. These challenges are particularly acute in Türkiye’s mountainous regions, where transportation and healthcare infrastructure are constrained. Similarly, the prominence of “family physician home care” in Yalova (100.0) may partly reflect the province’s relatively high aging population (10.2% aged 65+, compared to the national average of 10.6%) [19]. These localized trends suggest that regional health needs are shaped by demographic and infrastructural variables and must be addressed through place-specific interventions. These regional patterns are observational and cannot be causally attributed to specific healthcare needs without validation against utilization data.

Conversely, higher search rates for “switch family physician” in İstanbul (92.0) and Kırklareli (100.0) may point to dissatisfaction with existing services or higher expectations for provider choice in more urbanized settings. Meanwhile, widespread use of Central Physician Appointment System-related queries in cities such as Kütahya and Bilecik indicates strong engagement with digital health platforms, possibly due to better internet infrastructure and higher digital literacy. The lower search intensities observed in Eastern and Southeastern Türkiye do not necessarily indicate a reduced need for primary care, but rather underscore a "digital shadow" - where lower digital literacy and infrastructural constraints create a layer of digital vulnerability, potentially masking the true healthcare demands of these populations in web-based datasets.

These spatial patterns reinforce concerns about digital health equity. Rural regions, such as Ordu, with high on-call demand, suffer from poor healthcare access and limited digital infrastructure, creating digitally vulnerable populations [20]. In contrast, urban centers such as İstanbul enjoy better platform access but still exhibit signs of dissatisfaction. Such disparities suggest that digital tools can both mitigate and amplify health inequities, depending on contextual factors such as digital literacy and regional investment.

Among the 54 analyzed keywords, 28 (51.9%) showed no significant long-term trend after FDR correction, suggesting stable rather than growing public interest in several appointment- and logistics-related queries. Separately, 15 candidate keywords were excluded before analysis due to persistently low search volumes throughout the study period. These included procedure-specific queries, such as cancer screening, diabetes monitoring, and telehealth, suggesting that clinical service details are not actively sought through general web searches or that this information is obtained through direct provider contact. Overall, this pattern may indicate a potential communication gap; however, while public interest in family medicine as a concept appears to be increasing, engagement with its specific clinical scope through digital search behavior seems comparatively limited.

The observed search patterns identify areas of potential need and public interest that may inform primary care policy and planning. The trend, seasonal, and regional analyses provide descriptive findings. Interpretations linking these patterns to unmet need or system dissatisfaction are working hypotheses that require confirmation through clinical data. Nevertheless, these findings may offer preliminary indications that could be taken into account in broader policy and healthcare planning contexts. To address these disparities, the following policy-relevant directions are proposed as hypothesis-driven implications pending empirical validation. For example, mobile clinics equipped with telemedicine tools could provide care in underserved rural areas. Given Türkiye’s near-complete electronic health record adoption [21], integrating telehealth into routine practice is feasible and aligned with the World Health Organization (WHO)’s global strategy on digital health [22]. In addition, simplified, multimedia guides to the Central Physician Appointment System and e-nabız could improve accessibility for individuals with low literacy. Seasonal demand spikes - particularly in winter - could also inform staffing and resource allocation in family health centers.

Public education campaigns may focus on clarifying the role and capabilities of family physicians. Addressing frequently searched questions such as “what is a family physician?” and “can a family physician prescribe medication?” can help build public trust and encourage appropriate utilization of primary care. Improving digital health literacy, particularly in Türkiye’s eastern and rural regions, could also be prioritized to ensure equitable engagement with health technologies [20].

Türkiye’s experience highlights both the opportunities and challenges of integrating digital platforms into primary care. As other middle-income countries face similar gaps in digital infrastructure and regional equity, Türkiye’s model offers a useful case for global health systems aiming to build resilient, inclusive primary care services. Aligning with the WHO’s digital health goals, strategies, such as telehealth expansion, mobile clinics, and literacy campaigns, can improve both health outcomes and system responsiveness.

Limitations

This study has several limitations. As an ecological study, findings reflect population-level patterns and cannot be attributed to individual behaviors. Ecological fallacy - drawing individual-level conclusions from aggregate data - is a relevant concern when interpreting regional findings. Additionally, Google Trends provides normalized search data (0-100) rather than absolute search volumes, limiting direct comparisons between keywords. Google Trends does not capture user intent. The same search term may reflect health concern, professional interest, or casual curiosity, which limits the interpretability of individual keyword trends. Search data reflect interest rather than actual healthcare utilization, and thus should not be interpreted as definitive measures of service use. Cross-referencing these findings with MHRS appointment records or e-nabız platform data would strengthen the validity of the observed patterns. Additionally, Google Trends does not provide demographic information, which restricts deeper analyses of subpopulation behavior. Populations in regions with low digital access, particularly in eastern Türkiye, may be underrepresented, potentially skewing results [20]. While this study relies on digital proxies, the utility of search engine data has been validated in public health literature as a reliable reflection of official healthcare utilization statistics and disease surveillance trends, providing a real-time supplementary lens to traditional clinical registries.

Future research should build on these trend findings, while low-cost and easily accessible, by incorporating demographic data, exploring user intent behind search terms, and correlating search trends with health policy changes or public health events. Digital epidemiology thus holds significant promise for shaping evidence-based, equitable health systems in Türkiye and globally. Addressing these disparities requires a dual approach: enhancing digital infrastructure to capture "invisible" rural demands while simultaneously clarifying the clinical boundaries of family medicine to reduce informational noise in urban search behaviors.

## Conclusions

This study demonstrates that internet search query data can serve as a supplementary, scalable tool for monitoring public interest and identifying regional and seasonal variations in primary care engagement. Our findings reveal that while public interest in family medicine has steadily grown - accelerated by the pandemic's shift toward digital platforms - persistent queries regarding physicians' duties indicate ongoing information gaps about the clinical scope of the specialty. Geographic disparities in search behavior further point to a potential "digital shadow" in rural regions, suggesting localized informational deficits and health needs that require future confirmation through actual healthcare utilization data.

Rather than serving as definitive indicators of clinical demand, these digital footprints provide hypothesis-generating insights. Healthcare administrators and policymakers may consider utilizing such observational data as an additional resource to inform regional planning, anticipate seasonal resource allocation, and guide digital health literacy initiatives, thereby supporting the broader goal of equitable primary care delivery. 

## Appendices

A total of 56 keywords entered the quantitative analysis. Of these, 54 keywords were evaluated in the primary trend, seasonal, and pandemic-phase analyses (Tables 1-3). These keywords, translated from Turkish, included: "what is a family physician?," "family physician salary," "can a family physician prescribe medication?," "family health center," "what does a family physician do?," "family physician health report," "family medicine specialist," "MHRS appointment," "family physician MHRS appointment," "MHRS," "family physician e-devlet," "how to get an appointment family physician?," "health center working hours," "health center," "family physician," "how to become a family physician?," "family health center appointment," "family physician examination," "family physician on-call," "family medicine blood work," "family medicine," "family doctor appointment," "nearest family physician," "family physician e-nabız," "does the health center administer injections?," "family physician sick-leave report," "what tests are performed at the health center?," "family physician baby vaccination appointment," "family physician fee/is it paid?," "family physician pregnancy monitoring," "family physician online examination," "family physician appointment," "how to change family physician?," "family doctor e-nabız," "health center appointment," "family physician home care," "family physician duties," "family physician appointment cancellation," "family medicine emergency situation," "family doctor," "family physician remote examination," "family physician COVID test," "family physician quarantine report," "family physician pediatric examination," "family physician drug prescription," "family medicine system," "change family physician," "family medicine appointment," "search family physician," "family medicine specialization," "family physician address," and "FHC appointment."

Additionally, two comparative terms - "Internal medicine specialist" and "Internal medicine" - were included solely for the OLS regression analysis to compare search trajectories between family medicine and internal medicine.

A total of 15 candidate keywords were eliminated from the final analysis. Fourteen of these terms were excluded due to persistently insufficient search volumes (sparse Google Trends data, RSV ≈ 0) across the study period. These terms included: "family physician cancer screening," "family physician diabetes monitoring," "family physician heel prick test," "family physician smear test," "family physician chronic disease management," "family physician telehealth," "is the family physician open at night?," "family physician newborn registration," "find family physician," "family physician tele-examination," "doctor appointment health center," "family physician online appointment," "family physician query with TC ID," and "family physician vaccination appointment" (which was retained in the preliminary list but removed post-hoc upon verifying insufficient volume). One additional keyword, "neighborhood doctor," was excluded before analysis due to its status as outdated terminology that does not accurately reflect the modern family medicine system in Türkiye.
